# The Biotics Family: Current Knowledge and Future Perspectives in Metabolic Diseases

**DOI:** 10.3390/life12081263

**Published:** 2022-08-19

**Authors:** Codrina-Madalina Palade, Georgiana-Anca Vulpoi, Radu-Alexandru Vulpoi, Vasile Liviu Drug, Oana-Bogdana Barboi, Manuela Ciocoiu

**Affiliations:** 1 “Socola” Institute of Psychiatry, 700282 Iasi, Romania; 2 Neurology Department I, “Prof. Dr. N. Oblu” Emergency Clinical Hospital, 700309 Iasi, Romania; 3 Institute of Gastroenterology and Hepatology, “Saint Spiridon” Country Clinical Emergency Hospital, 700111 Iasi, Romania; 4 Department of Gastroenterology, Faculty of Medicine, “Grigore T. Popa” University of Medicine and Pharmacy, 700115 Iasi, Romania; 5 Department of Pathophysiology, Faculty of Medicine, “Grigore T. Popa” University of Medicine and Pharmacy, 700115 Iasi, Romania

**Keywords:** gut microbiota, metabolic diseases, biotics family, prebiotics, probiotics, postbiotics

## Abstract

Globally, metabolic diseases such as obesity, type 2 diabetes mellitus and non-alcoholic fatty liver disease pose a major public health threat. Many studies have confirmed the causal relationship between risk factors and the etiopathogenesis of these diseases. Despite this, traditional therapeutic management methods such as physical education and diet have proven insufficient. Recently, researchers have focused on other potential pathways for explaining the pathophysiological variability of metabolic diseases, such as the involvement of the intestinal microbiota. An understanding of the relationship between the microbiome and metabolic diseases is a first step towards developing future therapeutic strategies. Currently, much attention is given to the use of biotics family members such as prebiotics (lactolose, soy oligosaccharides, galactooligosaccharides, xylooligosaccharides or inulin) and probiotics (genera *Lactobacillus*, *Bifidobacterium*, *Lactococcus*, *Streptococcus* or *Enterococcus*). They can be used both separately and together as synbiotics. Due to their direct influence on the composition of the intestinal microbiota, they have shown favorable results in the evolution of metabolic diseases. The expansion of the research area in the biotics family has led to the discovery of new members, like postbiotics. In the age of personalized medicine, their use as therapeutic options is of great interest to our study.

## 1. Introduction

In recent years, the prevalence of metabolic diseases in developed countries has grown exponentially. This increase is associated with dynamic changes in dietary intake of macronutrients in recent decades. Based on national and international statistics, but also from a public health point of view, traditional approaches such as physical education and diet have not been successful in decreasing the prevalence of metabolic diseases. Therefore, recent research has focused on discovering new therapeutic methods to address this global public health problem. Pathogenic mechanisms linking the gut microbiota to metabolic diseases have been discovered and shown to play an important role in their development. Given the relationship between the gut microbiota and the pathogenesis of metabolic diseases, several therapeutic strategies have been developed to change the composition of the gut microflora, by using the biotics family [1,2].

## 2. Human Gut Microbiota: History and Present

Until a few decades ago, everything we knew about the gut microbiota was largely about its potentially pathological quality. In recent years, a growing number of metagenomic analyses have provided much more detailed information about the components of the gut microbiota, from their identification based on genetics to their role in different metabolic activities or in the pathogenesis of various diseases. These include intestinal diseases such as inflammatory bowel disease, irritable bowel syndrome or colorectal cancer, to metabolic diseases such as type 2 diabetes mellitus, non-alcoholic fatty liver disease, obesity or even Alzheimer’s disease, Parkinson’s disease or autoimmune diseases [3,4].

### 2.1. The Begins of Human Gut Microbiota

In the Western world, the first information about the gut microbiota originated around 1680, when Antonie van Leeuwenhoek examined his own feces under a microscope and found “more than 1000 animalcules”. Although his information was in circulation, it was only at the end of the 19th century that this field of research really took off [5]. Another description of gastrointestinal bacteria is given by John Goodsir of Edinburgh around 1842, when he found a microorganism called *Sarcina ventriculi* in the fluid expelled from the stomach of a 19-year-old man. In the 19th century, there were many researchers who debated the presence of microorganisms in the human gut [5]. In 1867, Lionel Smith Beale highlighted the presence of microorganisms in the stomach, intestines and human stools. Two years later, botanist Ernst Hallier discovered a large number of bacteria in the feces of healthy men. In 1881, research by german doctor Uffelmann revealed the presence of microorganisms in the feces of breastfed babies [5].

Research on the gut microbiota continued with Theodor Escherich, who in 1885 presented the first descriptions of a bacterial population found in the feces of healthy infants. He called it *Bacterium coli commune*, and later this bacterium was named after him, becoming one of the most famous bacteria: *Escherichia coli*. Another of Escherich’s papers that marked an important point in the understanding of the concept of the human microbial flora is “*The intestinal Bacteria of the infant and their relation to the physiology of digestion*”, where he details the intestinal bacterial composition of infants, the role of bacteria in the breakdown of food and the clinical implications of the intestinal flora [6].

Another name that is remarkable in the field of gut microbiota studies is Alfred Nissle. Interested in the interactions between different bacteria within the host, the German microbiologist realized that some strains of *Escherichia coli* in the normal gut flora prevent the growth of *Salmonella* and other enteropathogens. In the summer of 1917, during the First World War, when dysentery cases had reached a peak, Nissle noticed that a corporal seemed immune to the disease and suspected, based on his research, that this soldier’s flora contained a strain of *Escherichia coli* strongly antagonistic to intestinal pathogens. Nissle grew this specific strain of *Escherichia coli* in the laboratory, put it in gelatin capsules and patented it under the name *Mutaflor*, and it is still used today. The strain was later named *Escherichia coli Nissle 1917* [7,8].

The works of Antonie van Leeuwenhoe, John Goodsir of Edinburgh, Lionel Smith Beale, Theodor Escherich and Alfred Nissle laid the scientific foundations and advanced the clinical applications of microorganisms in the gut microbiota. However, due to the existing cultivation procedures at that time, knowledge about the involvement of the intestinal microbiota in human homeostasis was limited. A century later, technological developments highlight the complexity of the human intestinal microbiota and its importance in the pathophysiology of many diseases.

### 2.2. The Composition of the Human Intestinal Microbiota

The gut microbiota is considered the “invisible organ” of the human body. Although as humans, we are similar in terms of our genome, due to the diversity of our microbial community, we differ greatly. As well as the diversity of microbiota within an individual, called *alpha diversity*, there is a great deal of diversity in the microbial composition between individuals, called *beta diversity* [9,10]. Distinct populations of microorganisms belonging to the three domains of life were observed: *Archaea*, *Eukarya* and *Bacteria*. *Eukarya* comprises organisms that are complex structures, being surrounded by membranes. Regarding the *Archaea* domain, the gastrointestinal tract is colonized by *Methanobrevibacter mithii*. The bacterial population, which occupies the dominant place in the composition of the intestinal microbiota, develops rapidly from birth to the age of 2–3 years, when the adult-like composition is established [10].

Taxonomically, bacteria are classified by phylum, class, order, family, genus and species. The most representative phyla of the human intestinal microbiota are: *Bacteroidetes*, *Firmicutes* and *Actinobacteria* which represent approximately 98% of the intestinal microbiota. *Proteobacteria*, *Fusobacteria*, *Cyanobacteria* and *Verrucomicrobia* are the less present phyla, representing only 2%. The *Firmicutes* phylum is composed of over 200 different genera such as *Bacillus*, *Lactobacillus*, *Clostridium*, *Enterococcus* and *Ruminicoccus*. *Bacteroidetes* is composed of predominant genera such as *Bacteroides* and *Prevotella*. The phylum *Actinobacteria* is mainly represented by the genus *Bifidobacterium* [11,12].

**Figure 1:**
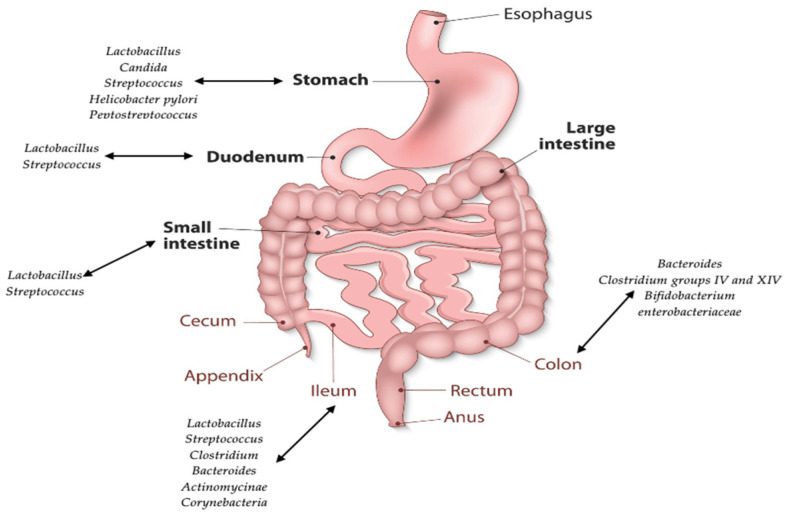
Distribution of the human gut microbiota.

The microbiota of the human gastrointestinal (GI) tract is a complex, dynamic and relatively stable community over time. However, it varies according to anatomical regions of the digestive tract, in that these regions differ in physiology, pH, O_2_ concentration, digestive flow and substrate availability. The O_2_ concentration and the pH undergo a gradual change along the GI tract. If the pH in the stomach is acidic and the medium is aerobic, the two parameters tend to change, resulting in a neutral pH and anaerobic medium in the large intestine [9,12]. As a result of the influence of the listed factors, bacteria have a specific distribution. The stomach has the lowest bacterial loads, especially *Lactobacillus*, *Streptococcus*, *Helicobacter pylori* and *Peptostreptococcus species*, due to unfavorable hosting conditions. In the small intestine there is an increase in microbial load from 10^2^–10^3^ (in the duodenum) to 10^4^–10^8^ (in the terminal ileum). Bacterial populations belonging to *Streptococcus* and *Lactobacillus* species are the dominant members of the small intestine, while *Clostridia*, *Streptococcus* and *Bacteroides* populations become the main residents of the large intestine, where there is an increase in load from 10^8^ in the caecum to 10^12^ bacteria/gram of fecal content (Figure 1) [9,11,12].

### 2.3. Human Gut Microbiota Variations

The gut microbiota varies in the same individual due to perinatal, age and environmental factors. The level of variation between individuals is mostly determined by enterotypes, body mass index levels and external factors such as lifestyle, exercise, ethnicity and dietary habits [11].

#### 2.3.1. Perinatal Conditions

The method of birth has an important impact on the development of an individual’s microbial community. If the baby is born vaginally, it comes into contact with the mother’s vaginal and intestinal microbiota, where Lactobacillus, Prevotella, Bifidobacterium longum and Bifidobacterium catenulatum, but also facultative anaerobic bacteria such as Escherichia coli, Staphylococcus, Bacteroides fragilis and Streptococcus predominate. On the other hand, a newborn baby by caesarean section does not come into contact with the mother’s vaginal and intestinal microbiota, and its microbiota composition will be made up of microorganisms from the environment and from the mother’s skin. Staphylococcus, Corynebacterium, Propionibacterium, but also Clostridium cluster I and Clostridium difficile members were observed in these newborns [13,14,15]. Another factor that influences the composition of the intestinal microbiota in newborns is the method of feeding them. It has been observed that infants who are fed powdered milk are colonized with Escherichia coli, Bacteroides and Clostridium difficile. Breastfed infants have high levels of bifidobacteria, especially *B. breve*, *B. bifidum*, *B. longum* and *B. adolescentis* and lactobacilli, and a low number of pathogens compared to infants fed powdered milk that have a diverse microbial flora where staphylococci, enterococci, enterobacteria predominate [11,13].

#### 2.3.2. Age

The composition of the gut microbiota also differs depending on the age of the person. At one year old, the composition of a child’s microbial flora shows an abundance of *Akkermansia muciniphila*, *Bacteroides*, *Veillonella*, *Clostridium coccoides* spp. and *Clostridium botulinum* spp. Microbiota diversity increases with age until it becomes a stable composition in adults, where the three phyla dominate: *Firmicutes*, *Bacteroidetes* and *Actinobacteria*. But with advancing age, the gut microbiota undergo changes due to the action of various external factors [11].

#### 2.3.3. Enterotypes

Everyone has a gut microbiota that is characterized by a group of bacteria, called enterotypes: *enterotype I* where *Bacteroides* predominates, *enterotype II* where *Prevotella* predominates and *enterotype III* where *Ruminococcus* predominates. Although each enterotype does not represent a clear identity, the groups of bacteria characteristics of each enterotype have different functional characteristics. For example, members of *enterotype I* obtain energy from carbohydrates via the glycolysis and pentoxophosphate pathways, while bacteria belonging to *enterotypes II* and *III* are capable of degrading the mucin glycoproteins of the intestinal mucosal layer [11,16].

#### 2.3.4. Exercise

There are studies showing a link between exercise frequency and gut microbiota composition. Sport increases gut microbial diversity, especially among *Firmicutes* (*Clostridiales*, *Roseburia*, *Lachnospiraceae* and *Erysipelotrichaceae*). Another study highlighting the involvement of exercise in microbiota composition is a study on rugby players whose gut microbiota recorded a high diversity of *Firmicutes*, in particular *Faecalibacterium prausnitzii* [11,17]. Also, the presence of *Akkermansia muciniphila* is more abundant in athletes than in those who do not practice sports [18].

#### 2.3.5. Diet

After childhood, the intestinal microbiota continues to develop, and this process is continuously influenced by diet, which is a key element in defining the shape, structure and various intestinal microflora. There are several types of diet that can influence the microbiota: Mediterranean, vegetarian and Western diet. Of the three types of diet, the vegetarian and Mediterranean influences the microbiota positively, while the Western diet negatively influences the composition of the intestinal microbiota. Being low in animal protein and saturated fat, increased levels of *Prevotella* and *Firmicutes* were observed in patients on a Mediterranean diet [19,20]. People who have adopted a Mediterranean diet have an increase in the production of short-chain acids (SCFA) and anti-inflammatory properties that reduce the occurrence of chronic inflammatory diseases such as type 2 diabetes mellitus [20].

## 3. Gut Microbiota and Metabolic Diseases

The link between gut microbiota and metabolic diseases has led to the investigation of microbial communities in people with obesity, type 2 diabetes mellitus or non-alcoholic fatty liver disease. Evidence has shown that there are differences between the microbial composition of healthy people and that of people with the metabolic diseases listed above [1,2].

### 3.1. Gut Microbiota Composition in Metabolic Diseases

In terms of gut microbiota composition in people with obesity, changes were observed at the filum level, as well as at a more specific level such as species or class. There are currently numerous studies showing a difference between the gut microbiota composition of individuals with obesity and normal body weight individuals. In general, a change in the *Firmicutes*/*Bacteroidetes* ratio was observed, in the sense of an increase of the former phylum [21].

A first observation was made by Ley and co-workers who studied the gut microbiota composition of both lean wild-type (+/+) or heterozygous (ob/+) mice and ob/ob mice. The results showed that the phyla *Fimicutes* (60–80%) and *Bacteroidetes* (20–40%) were the most abundant. It was also observed that mice homozygous for the aberrant leptin ob/ob gene showed different levels of bacteria compared to the other control groups. Ob/ob mice showed a 50% decrease in the *Bacteroidetes* population and a proportional increase in *Firmicutes* [21,22]. A difference in gut microbiota composition was also found in lean and obese Ossabaw and Göttingen minipigs. It was observed that obese Ossabaw minipigs had lower levels of *Prevotella* and *Lactobacillus* genera and higher levels of *Clostridium* compared to lean Ossabaw minipigs. Obese Ossabaw minipigs also had higher levels of Firmicutes and a lower abundance of *Bacteroidetes* compared to lean Ossabaw minipigs. Differences were also observed in Göttingen pigs. The level of *Clostridium* XIV group was 7.6 times higher in the cecal microbiota of obese Göttingen minipigs compared to lean ones [23].

The human gut microbiota also appears modified in obesity. An interesting study highlighting the difference in gut microbiota composition between obese and normal body weight individuals is that conducted by Hao-Jiang Zuo and collaborators. They found a lower amount of *Clostridium perfringens* in people with obesity compared to those with normal body weight. They also observed a higher amount of *Enterococci* in the group of obese people than in the group of normal-weight people [24,25].

Goodrich et al. demonstrated in their study that a specific bacterial taxon is associated with obesity, namely *Christensenellaceae* spp., and has been proposed as a microbial biomarker for obesity [26,27].

Million and his collaborators, in their study of 263 individuals, including 134 individuals with obesity, 38 overweight individuals, 76 lean individuals, and 15 individuals with anorexia, examined various correlations between bacterial species and obesity, being overweight or anorexia. Thus, they observed that there was a higher prevalence of *Lactobacillus* in individuals with obesity and overweight individuals compared to lean individuals, as well as an increased prevalence of *Methanobrevibacter smithii* in lean individuals compared to individuals with obesity and overweight individuals [28].

Changes in gut microbiota composition associated with obesity have also been observed in children. In a study of 84 fecal samples from 30 children with obesity, 24 overweight children and 30 lean children, the *Bacteroides fragilis* group was found to be present in higher concentrations in the children with obesity group and the overweight children group compared to the lean children group. Aditionally, feces from children with obesity and overweight children had higher amounts of *Lactobacillus* spp. than those from lean children. In contrast, *Bifidobacterium* spp. was found in higher numbers in the lean children group than in the children with obesity and overweight children group [29].

As with people with obesity, differences in gut microbiota composition have been observed in people with type 2 diabetes mellitus. A first observation was quantified by Larsen and co-workers who identified reduced proportions of *Firmicutes* and *Clostridia* and increased levels of *Betaproteobacteria* in people with type 2 diabetes mellitus [30].

Specific gut microbiota profiles have also been identified by Kristine H. Allin and collaborators. Thus, they observed that the genus *Clostridium* had a low abundance while *Dorea* (*Ruminococcus*), *Sutterella* and *Streptococcus* were increased in people with diabetes compared to those with normal glycemic control. Additionally, members of the order *Clostridiales* and *Akkermansia muciniphila* showed low abundance in people with prediabetes [31].

Moran Yassour and co-workers found that the decrease of *Akkermansia muciniphila* can be used as biomarker for the early diagnosis of type 2 diabetes mellitus [32]. Another potential biomarker of type 2 diabetes mellitus is *Verrucomicrobiae*. Zhang and his team observed a significant reduction of this bacterium in the early and advanced stages of people with type 2 diabetes mellitus. They also observed that butyrate-producing bacteria such as *Akkermansia muciniphila ATCCBAA-835* and *Faecalibacterium prausnitzii L2-6* showed a higher abundance in the normal glucose tolerance (NGT) group than in the prediabetes group [33]. Additionally, Qian Yang and his team suggested that *Acidaminococcus*, *Aggregatibacter*, *Anaerostipes*, *Blautia*, *Desulfovibrio*, *Dorea* and *Faecalibacterium* are associated with type 2 diabetes mellitus [34]. Another study conducted in a Swedish population highlights the link between gut microbiota composition and type 2 diabetes mellitus, where a decrease in butyrate producers such as *Pseudoflavonifractor* spp., *Clostridium* spp., *Alistipes* spp., *Faecalibacterium* spp. and *Oscillibacter* spp. is observed [35].

In a study involving 26 patients with type 2 diabetes, *Prevotella*, *Firmicutes*, *Proteobacteria* and *Bacteroidetes* were observed in increased abundance [36]. Xue Zhao and his collaborators compared the gut microbiota composition of people with type 2 diabetes mellitus and people with normal glycemic control in their study. Thus, 137 patients with type 2 diabetes mellitus and 179 people with normal glycemic control participated in their study. They found that the α-diversity of bacterial taxa in the type 2 diabetes mellitus group showed a significant decrease compared to the control group. At the genus level, *Bacteroides* and *Prevotella* decreased the most, while *Escherichia*, *Shigella*, *Lachnospiraceae*, *Enterococcus* and *Klebsiella* had different degrees of expansion in the type 2 diabetes mellitus group [37].

As mentioned above, the composition of the gut microbiota differs in people suffering from different diseases compared to healthy people. Differences in gut microbiota composition are also found in people with non-alcoholic fatty liver disease (NAFLD). In this regard, Boursiers and his collaborators conducted a study in which they included 57 patients with biopsy-proven NAFLD. Of these, 30 patients had stage F0/F1 fibrosis on liver biopsy (10 of whom had non-alcoholic steatohepatitic liver disease (NASH)) and 27 patients had significant F2 fibrosis (25 of whom had non-alcoholic steatohepatitic liver disease). It was observed that in patients with NASH and F2 *Bacteroides* abundance was significantly increased while *Prevotella* was decreased. Additionally, in F2 patients *Ruminococcus* was significantly higher [38].

Another study that highlights differences in gut microbiota composition in people with NAFLD is that conducted by Loomba and his team. They observed that people with NAFLD have increased levels of *Proteobacteria* and *Firmicutes*. They also found that depending on the stage of fibrosis, NAFLD patients have different concentrations of bacteria. Thus, in people with NAFLD and fibrosis stage below F2, increased levels of *Eubacterium rectale* and *Bacterioides vulgatus* were evident, while in people with NAFLD and fibrosis stage higher than F2, *Bacteroides vulgaris* and *Escherichia coli* dominated [39].

Del Chierico and his team have also done research into the composition of the gut microbiota. They observed that in patients with NAFLD, *Actinobacteria* showed an increase while Bacteroidetes decreased. An increase of *Bradyrhizobium*, *Anaerococcus*, *Peptoniphilus*, *Propionibacterium acnes*, *Dorea* and *Ruminococcus* and a reduction of Oscillospira and Rikenellaceae were observed in NAFLD patients compared to the control group [40].

Caussy et al. conducted a study in which they included participants with NAFLD and controls. They observed that in people with NAFLD there was an increase in *Enterobacteriaceae*, *Streptococcus*, *Gallibacterium* and *Megasphaera* [41]. Another study showing changes in the gut microbiota of people with NAFLD is by Lee et al. They observed that in people with NAFLD there is an increase in *Ruminococcaceae* and *Veillonellaceae* [42].

### 3.2. Gut Microbiota and Pathogenesis of Metabolic Diseases

The mechanisms by which gut microbiota influence the pathophysiology of metabolic diseases are numerous, but few have attracted attention. The gut microbiota modulates inflammation, interacts with dietary constituents andaffects intestinal permeability, glucose and lipid metabolism, choline and trimethylamine oxide metabolism, insulin sensitivity and energy homeostasis and endogenous ethanol production [2,21,43].

The gut microbiota causes the production of pro-inflammatory molecules such as lipopolysaccharides (LPS) and peptidoglycans, which interact with host metabolism. In principle, metabolic diseases can be correlated with low-grade inflammation [44]. The intestinal mucosa of the GI tract shows an important role in nutrient absorption and in maintaining the integrity of the intestinal barrier. It acts as a gate that allows the translocation of essential macronutrients and restricts the passage of bacteria, toxic molecules and luminal antigens, and the interaction of epithelial cells with the gut microbiota leads to the formation of a pre-epithelial film consisting of a layer of mucus-secreting Immunoglobulin A (IgA) molecules, immune cells, enzymes and metabolites of commensal bacteria. Epithelial integrity can be affected by changes in the distribution and location of zonule occludens-1 (ZO-1) and occludin, two tight junction proteins, in intestinal tissue [1,45,46].

The term metabolic endotoxemia was first observed in mice and described as the ability of bacterial lipopolysaccharides to cause a systemic inflammatory state by translocating bacteria into the bloodstream and which in combination with CD14 forms toll-like receptor ligand-4 (TRL4) [47,48]. Due to the fact that in metabolic diseases the intestinal permeability is altered, an increased absorption of LPS occurs, which induces systemic inflammation. LPS induces expression of pro-inflammatory cytokines mitogen-activated kinase-dependent kinase (MAPK) and nuclear factor KB (NF-KB) in human adipocytes. Increased plasma LPS levels have been correlated with induction of hyperphagia and obesity [46].

The *Faecalibacterium* and *Roseburia* genera provide protection against bacterial translocation and reduce intestinal permeability. Normally, *Roseburia intestinalis* induces the secretion of anti-inflammatory interleukin-10 (IL-10), a cytokine that contributes to improved glucose metabolism, as well as the secretion of interleukin-22 (IL-22), an anti-inflammatory cytokine known to restore insulin sensitivity. All these beneficial effects are not possible in type 2 diabetes mellitus (T2DM) because in T2DM the bacteria mentioned above are in significantly reduced quantities [2,49].

There is also low-grade inflammation in NAFLD. Kupffer cells, which express the highest levels of TRL-4 in the liver, are the primary cells in liver inflammation that respond to LPS by producing pro-inflammatory cytokines, chemokines and reactive oxygen species (ROS). The “second-hit” mechanism of NAFLD pathogenesis includes lipid peroxidation and ROS generation [50].

The gut microbiota produces several potentially hepato-toxic molecules, including ethanol, acetaldehyde, ammonia or phenols, which are transported to the liver via the portal system, where they are metabolized. Dysbiosis in NAFLD is associated with bacteria such as *Escherichia*, *Bacteroides*, *Bifidobacterium*, *Clostridium* and *Klebsiella pneumonia* which can produce alcohol [51]. It has been observed that high levels of endogenous ethanol are found in NAFLD patients compared to the control group. Endogenously produced ethanol is responsible for macrophage activation and the production of pro-inflammatory cytokines, by which bacteria can disrupt the intestinal barrier, thus stimulating translocation of endotoxins into the portal circulation. Acetaldehyde, a metabolite of alcohol, stimulates Kupffer cells, which in turn activates the immune system, thereby increasing nitric oxide production and cytokines such as tumor necrosis factor alpha, which further causes the production of reactive oxygen species [52].

Choline is an essential phospholipid with key roles in various physiological processes such as lipid metabolism, where it prevents the accumulation of abnormal lipids in the liver, second messenger transduction, enterohepatic circulation of bile acids and cholesterol metabolism. Choline deficiency stimulates hepatic steatosis. Gut microbiota secrete enzymes that catalyze the conversion of dietary choline into toxic methylamines (dimethylamine and trimethylamine) which are then taken up by the liver where they are converted to trimethylamine N-oxide, which causes liver inflammation. So dysbiosis of the gut microbiota can cause dual non-alcoholic fatty liver disease, both through decreased choline availability and increased toxic methylamines [50,52,53,54].

Amines and polyamines are fermentation products of commensal bacteria. They help form trimethylamine metabolized from choline, which is then transported to the liver, oxidized by flavin monooxygenase 3 and converted to trimethylamine oxide (TMAO), which plays an important role in the pathogenesis of type 2 diabetes mellitus [55]. In vivo studies have shown that bacteria such as *Firmicutes*, *Proteobacteria*, *Clostridium* and *Escherichia fergusonii*, are TMAO generators, and in T2DM these bacteria are increased [56].

## 4. The Biotics Family: Current Knowledge in Metabolic Diseases

**Figure 2:**
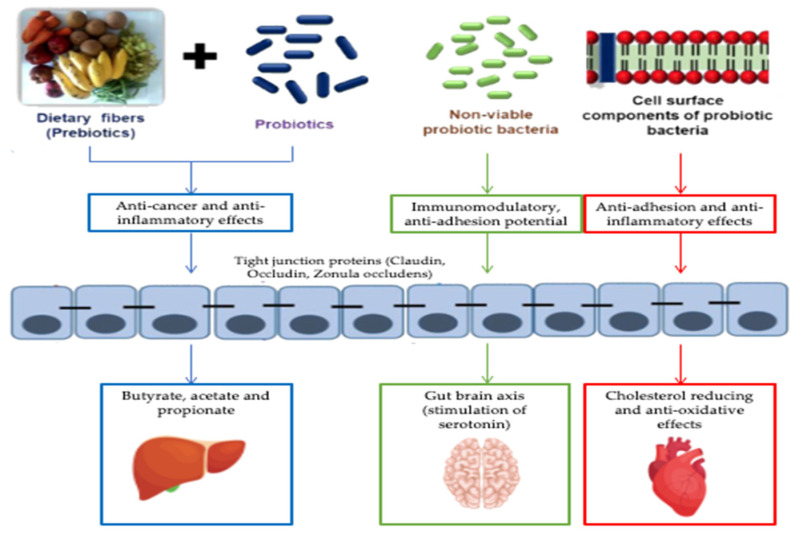
Effects of the biotics family on metabolic diseases (adapted to Nataraj et al. [58]).

The human intestine is a complex ecosystem wherein gut microbiota, nutrients and host cells work together to maintain homeostasis and host development. Because of this close relationship, any change in the composition of the gut microbiota can have serious consequences for the health of the host. It is therefore important to maintain a balanced gut microflora composition. In this sense, there is a great interest in the use of non-invasive and safe methods, such as the use of prebiotics or probiotics and more recently the use of postbiotics (Figure 2) [57].

### 4.1. Probiotics

The first use of the probiotic concept as a voluntary modification of the intestinal microbiota was in ancient China to treat infections or food poisoning [59]. But the era of probiotics began in the true sense in the early twentieth century, during the time of Ilia Ilyich Mechinikov, who correlated the longevity of Bulgarians with the high consumption of fermented milk containing lactic acid bacteria. The pioneers of the term probiotic were D.M. Lilly and R.H. Stillwell who described that substances secreted by a microorganism stimulate the growth of another microbe [59,60,61]. Currently, the latest definition of probiotics characterizes them as living microorganisms, which when administered in adequate amounts, provide the host with health benefits and which have the characteristic of surviving inside the body without the ability to transfer pathogenic elements, antibiotic resistance and toxicity [62].

For a preparation to be considered an ideal probiotic, it must meet several criteria: (1) it must be resistant to both the acidic pH of the stomach and the alkaline pH of the other components of the gastrointestinal tract, (2) it must be resistant to processing, (3) it must be of human origin, (4) it must be non-pathogenic, (5) it must have the ability to stimulate local metabolic activity and (6) it must stimulate the immune system [63]. Microorganisms that are used as probiotics are exemplified in Table 1. Sources of probiotics can be either of human origin, from the large intestine, small intestine or breast milk, or of animal origin from various foods such as yogurt, frozen yogurt, kefir, whipped milk, acidophilus milk, aged cheeses, fermented cabbage, pickles and olives produced by traditional methods [60,62,64].

#### 4.1.1. Mechanisms of Action of Probiotics

**Figure 3:**
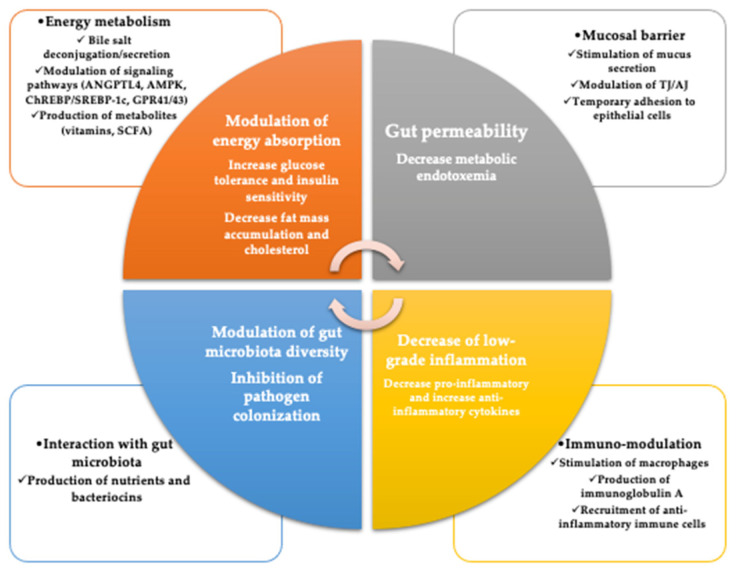
Potential beneficial effects of probiotic against metabolic disorders (GPR, G protein-coupled receptor; SCFA, short-chain fatty acid; ChREBP, carbohydrate-responsive element-binding protein; SREBP, sterol regulatory element-binding protein; AMPK, AMP-activated protein kinase; ANGPTL4, angiopoietin-like protein 4; TJ, tight junction; AJ, adherens junction) (adapted to Le Barz et al. [66]).

The mechanisms by which probiotics work are numerous, but some of them are known. These include improving the epithelial barrier, increasing adhesion to the intestinal mucosa, and concomitant inhibition of pathogen adhesion, competitive exclusion of pathogenic microorganisms, production of antimicrobial substances and modulation of the immune system (Figure 3) [65].

The gastrointestinal epithelium performs many important functions, including establishing a physical barrier between the external environment and the host’s immune system, maintaining the integrity of the digestive tract. As a result, the integrity and function of this barrier allow nutrients and beneficial molecules to pass through, protecting the host from harmful substances. This integrity is given by tight epithelial junctions (TJs), which represent multiprotein complexes composed of transmembrane proteins, (such as F-actin, zonula occludens—ZO, junction molecule—JAM, claudins, myosin II and tricellulin) that interact extracellularly with neighboring tight epithelial junctions and intracellularly connects to the cell cytoskeleton. When there is a disorder in these proteins, the functionality of the physical barrier is compromised and thus the intestinal permeability appears, which is responsible for the development of many pathological disease [67].

A study highlighting the effect of *Escherichia coli Nissle 1917* (EcN) was performed on germ-free mice, which were colonized with EcN, and it was observed that this colonization led to overexpression of ZO-1, thus stabilizing the tight epithelial junctions [65,67,68]. Another probiotic that influences the properties of the intestinal epithelium is *Lactobacillus rhamnosus*. This probiotic has protective effects against enterohemorrhagic infections with *Escherichia coli O157: H7*. Johnson-Henry and co-workers found that epithelial cells that had been treated with probiotics before *E. coli* infection had higher levels of ZO-1 expression than those infected with *E. coli* [67,69]. The intestinal epithelium is covered by a layer of viscoelastic mucus that performs functions such as: a barrier against harsh light environment, facilitating the passage of food, preventing the firm adhesion of bacteria to epithelial cells, stopping their passage in the lamina propria. The layer of mucus in the gastrointestinal tract is produced by mucins. These are high molecular weight glycoproteins, being divided into two groups: secretory mucins (MUC2, MUC5AC, MUC5B and MUC6), being responsible for the formation of the mucus layer and transmembrane mucins (MUC1, MUC4, MUC13, MUC16) [65,67].

Mucin expression is regulated by probiotic bacterial strains. For example, the *Lactobacillus plantarum 299v* strain inhibits the adhesion of enteropathogenic *Escherichia coli* to the intestinal epithelial cell line HT-29. Incubation of *L. plantarum strain 299v* with HT-29 results in increased mRNA expression of MUC2 and MUC3 genes, emphasizing that this probiotic stimulates epithelial cells to secrete mucins that decrease the binding of enteric pathogens to intestinal epithelial cells [67,70]. The probiotic *Escherichia coli Nissle 1917* also increases the expression of mucin genes. Incubation of HT-29 cells with *E. coli Nissle 1917* shows an increased growth of MUC2, MUC3, MUC5AC and MUC5A genes [67]. Another probiotic that increases mucin expression is *Lactobacillus casei,* which inhibits the translocation of pathogenic bacteria and increases the MUC2 gene expression [67,71].

Another property of probiotics is the production of antimicrobial substances. They can protect the host against infectious bacteria and promote the survival of the common ones. The most representative probiotics that have antimicrobial characteristics are those of the *Lactobacillus* class [67]. An example is the *Lactobacillus brevis strain 925A* which influences the immune system by producing a bacteriocin called *brevicin 925A*. It is effective against *Listeria monocytogenes* and *Streptococcus mutans*, which are responsible for food poisoning and dental caries [72].

#### 4.1.2. Probiotics and Metabolic Diseases

In recent years, scientific research has shown the beneficial effects of probiotics in clinical practice. There are studies that highlight the positive role of probiotics in various diseases, such as metabolic diseases.

Yukio Kadooka and his colleagues show in a randomized controlled study of 210 Japanese people that a low-dose consumption of *Lactobacillus gasseri 2055* has a significant effect of lowering abdominal adiposity in adults by an average of 28.5% [60,73]. In a three-week study involving 35 subjects, 25 of whom had a 1500 kcal/day diet supplemented with cheese containing the probiotic *Lactobacillus plantarum TENSIA* and the other 15 with control cheese, highlighted a significant decrease in body mass index in the group of those with probiotic cheeses [60,74]. In a randomized, double-blind, controlled study, 75 healthy people were randomized to groups who received plain yogurt on a low-calorie diet, or who received probiotic yogurt on a low-calorie diet, or who received yogurt with probiotics and without a low-calorie diet for about 8 weeks. In the group that received a low-calorie diet with yogurt containing probiotics such as *Lactobacillus acidophilus La5*, *Bifidobacterium BB12* and *Lactobacillus casei DN001*, a reduction in body mass index, fat percentage and leptin levels was observed [75,76].

Type 2 diabetes mellitus is associated with intestinal dysbiosis, and the administration of probiotics may be a way to restore the intestinal microbiota in these patients. In a double-blind, randomized, placebo-controlled study, 50 volunteers with type 2 diabetes mellitus consumed fermented milk (120 g/day) daily for 6 weeks. They were divided into two groups: the group that consumed fermented milk containing *Lactobacillus acidophilus La-5 and Bifidobacterium animalis subsp lactic BB-12,* and the control group, which consumed conventional fermented milk. After 6 weeks, a significant decrease in hemoglobin A1c levels was observed in the group that consumed milk containing probiotics [76,77]. Another study that investigated the effects of probiotic and conventional yogurt on lipid profile in people with type 2 diabetes mellitus was performed on a group of 60 volunteers who received for 6 weeks 300 g of probiotic yogurt containing *Lactobacillus acidophilus La5* and *Bifidobacterium lactis Bb12* or 300 g of conventional yogurt. At the end of the 6 weeks, it was observed that the consumption of yogurt with probiotic led to a decrease of 4.54% of total cholesterol and a decrease of 7.45% of LDL-c [76,78].

As we know, oxidative stress is a key element in the pathogenesis of type 2 diabetes mellitus. The beneficial effects of probiotics on patients with type 2 diabetes mellitus were highlighted in a study involving 64 patients, who were divided into two groups: a group receiving 300 g of probiotic yogurt containing *Lactobacillus acidophilus La5* and *Bifidobacterium lactis Bb12* and a group receiving 300 g/day of conventional yogurt for 6 weeks. After 6 weeks, in the group that had consumed probiotic yogurt, there was a significant decrease in youth blood glucose and hemoglobin A1c [76,79].

Another pathology where the beneficial effect of probiotic use is highlighted is non-alcoholic fatty liver disease. A study in this regard was performed on 20 children with obesity who had persistent hypertransaminasemia and ultrasonographic hyper-reflexogenic liver, where for 8 weeks they received either the probiotic *Lactobacillus rhamnosus GG* strain or placebo. After 8 weeks of treatment, there was a significant decrease in alanine aminotransferase in the probiotic group. Therefore, the probiotic strain of *Lactobacillus rhamnosus* can be considered as a therapy for non-alcoholic fatty liver disease [80]. Another randomized, double-blind, controlled clinical trial in 72 patients with non-alcoholic fatty liver disease (NAFLD) highlighted the beneficial effects of probiotics. This study included 72 people with NAFLD who consumed for 8 weeks 300 g/day of probiotic yogurt containing *Lactobacillus acidophilus La5* and *Bifidobacterium lactis Bb12* or conventional yogurt. In the group of those who consumed probiotic yogurt, a reduction in serum levels of alanine aminotransferase, aspartate aminotransferase, total cholesterol and, respectively, low-density lipoprotein cholesterol by 4.67, 5.42, 4.1 and 6.92% was observed [81].

In another randomized trial, significant improvement was shown in children with NAFLD after the use of probiotics. A group of 48 randomized children (only 44 completed the study) used VSL#3 (VSL#3, a mixture of eight probiotic strains (*Streptococcus thermophilus*, bifidobacteria [*B. breve*, *B. infantis*, *B. longum*], *Lactobacillus acidophilus*, *L. plantarum*, *L. paracasei*, and *L. delbrueckii* subsp. *bulgaricus*), and placebo for 4 months. In the group consuming VSL#3, positive changes in liver architecture objectified by ultrasonography were observed, as well as a decrease in body mass index and an increase in GPL-1 [82]. Sang Bong Ahn and co-workers investigated the effects of probiotic treatment on visceral fat area (VFA) and intrahepatic fat fraction (IHF) in patients with non-alcoholic fatty liver disease. In their study, 68 obese patients with NAFLD were included and randomly assigned into two groups: a group receiving a probiotic mixture with six bacterial species (*Lactobacillus acidophilus*, *L. rhamnosus*, *L. paracasei*, *Pediococcus pentosaceus*, *Bifidobacterium lactis*, and *B. breve*) and a group receiving placebo for 12 weeks. A decrease in weight and a reduction in IHF and triglycerides was observed in the group receiving the probiotic mixture [83].

Nazarii Kobyliak and collaborators conducted a double-blind study in which they highlighted the superior effects of probiotics compared to placebo in type 2 diabetes mellitus patients with NAFLD. A total of 58 patients were divided into two groups: one receiving the probiotic “Symbiter” (consisting of 14 genera of probiotic bacteria *Bifidobacterium*, *Lactobacillus*, *Lactococcus*, *Propionibacterium*) and one receiving placebo, and the duration of the study was 8 weeks. As promising results, changes in fatty liver index (FLI) and liver stiffness (LS), as well as changes in aminotransferase activity and cytokine levels (TNF-α, IL-1β, IL-6, IL-8, and IFN-γ) were observed [84].

Supplementation with *Akkermansia muciniphila* has been shown to be effective in preventing or even treating metabolic disorders, including obesity. In this regard, Yang and his collaborators have highlighted its anti-obesity properties. In a sample of mice fed a high-fat diet (HFD), they administered strains of *Akkermansia muciniphila* isolated from human stool samples six times a week for 12 weeks. The results were favorable, with improved glucose homeostasis and insulin sensitivity, with prevention of body weight gain and caloric intake. Effects such as inhibition of low-grade intestinal inflammation and restoration of damaged intestinal integrity, prevention of hepatic steatosis and improvement of liver function were also associated [85].

However, it was also observed that *A. muciniphila* shows beneficial effects and without affecting dietary intake, its administration significantly decreased body weight gain and total body fat index. Depommier and his team observed that after 5 weeks of administration of *A. muciniphila*, a decrease in body weight and a significant reduction in fat table were achieved in mice fed a normal diet [86,87].

### 4.2. Prebiotics

Although dietary oligosaccharides have long been used in Asia for health, prebiotics have been talked about since the early 20th century, when Retteger and Cheplin suggested that milk sugars contribute to the modification of the gut microbiota in humans to stimulate the growth of *Bacillus acidophilus* [88]. Over the years, studies have shown that certain nutrients stimulate the growth of beneficial bacteria in the gut.

A first definition of these nutrients was given by Glenn Gibson and Marcel Roberfroid in 1995 [88,89]. They described it as a non-digestible food ingredient that has a beneficial effect on the host by selectively stimulating the growth and/or activity of one or a limited number of bacteria in the colon, thereby improving the health of the host [88,89]. In 2003, the scope of prebiotics was extended to include the mouth, stomach, small intestine, vagina and skin. At that time, according to the definition, only some compounds from the carbohydrate group (such as fructo-oligosaccharides (FOS) and inulin), lactulose and galacto-oligosaccharides (GOS) could be classified as prebiotic. However, in 2008, the term prebiotic was improved to “a non-viable food component that results in specific changes in the composition and/or activity of the gastrointestinal microbiota, conferring a health benefit to the host”. A new, and latest, definition for prebiotic was given in 2017 when the International Scientific Association for Probiotics and Prebiotics (ISAPP) proposed that prebiotic is “a substrate that is selectively utilized by host microorganisms conferring a health benefit” [88,90].

However, the term prebiotic was attributed to several food components and therefore it was necessary to establish clear criteria for classifying an ingredient as a prebiotic. These criteria are: the prebiotic must be resistant to the acidic pH of the stomach and resist digestion in the upper digestive segments in order to reach the colon, where it meets the second criterion of being selectively fermented by microorganisms. This process leads to an increase in the abundance of various short-chain fatty acids, a moderate reduction in colonic pH, a reduction in nitrogenous end products or improved immunological function, all of which fall under the third criterion, the results being beneficial to the host. Another criterion is the selective stimulation of the growth of commensal microorganisms and the last criterion that a prebiotic must fulfil is its resistance to food processing conditions and its ability to remain unchanged, unaltered or undegraded [91,92,93].

Prebiotics are carbohydrates with different molecular structures, which are naturally present in human and animal food. These are considered as prebiotic: fructooligosaccharides (FOS), galactooligosaccharides (GOS), isomaltooligosaccharides (IMO), xylooligosaccharides (XOS), transgalactooligosaccharides (TOS), soy oligosaccharides (SBOS), pectin, inulin and lactulose [91]. Table 2 gives examples of prebiotic sources [94].

#### 4.2.1. Mechanisms of Action of Prebiotics

The functions of a prebiotic are not fully known, but several mechanisms have been proposed: (1) it regulates the action of hepatic lipogenic enzymes influencing the production of short chain fatty acids, (2) it controls mucin secretion, (3) it causes an increase of lymphocytes/leukocytes in gut-associated lymphoid tissues (GALT) and (4) it causes increased secretion of IgA by GALT which may stimulate the phagocytic function of intra-inflammatory macrophages [91]. Prebiotics can also inhibit the growth of pathogens and increase the absorption of minerals, especially magnesium and calcium [91].

#### 4.2.2. Prebiotics and Metabolic Diseases

**Figure 4:**
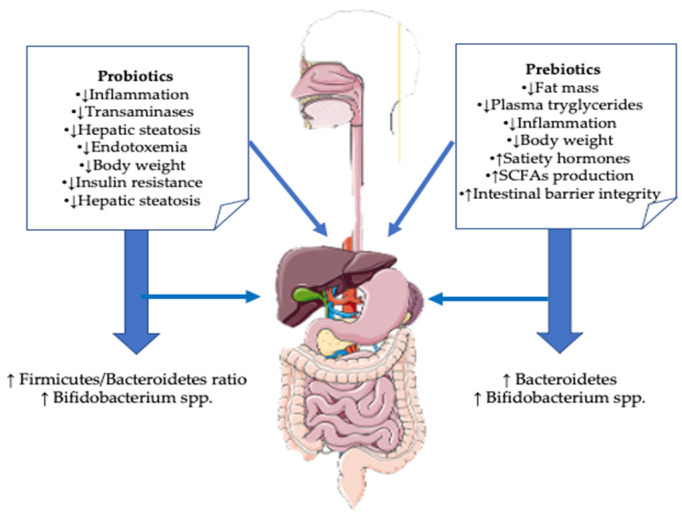
Effects of probiotics and prebiotics on NAFLD (adapted to Porras et al. [95].)

In recent years, prebiotics have been shown to have a remarkable influence on the health of the human body, making them potential therapeutic options to improve the quality of human life against many diseases, including metabolic diseases (Figure 4).

A randomized, double-blind study was performed in 48 adults with a body mass index greater than 25 kg/m^2^ who were divided into two groups: one group received placebo and one group received 21 g oligofructose for a period of 12 weeks. In the group of those who received oligofructose, a decrease in body weight and a glucose decrease was observed [96].

Another study where the beneficial effect of using a prebiotic is highlighted is represented by the study of Evelyne M. Dewulf and his collaborators. They conducted a double-blind, placebo-controlled intervention study involving 30 obese women. These were divided into two groups: one receiving inulin/oligofructose and another receiving placebo 16 g/day for 3 months. It was observed that in the group of those who received prebiotics there was an increase in *Bifidobacterium* and *Faecalibacterium prausnitzii*, both bacteria with a negative impact on serum lipopolysaccharides. A decrease in the levels of *Bacteroides intestinalis*, *Bacteroides vulgatus* and *Propionibacterium* was also observed [97,98].

Another study where the effectiveness of a prebiotic was highlighted, this time on a group of children, is that of Alissa C. Nicolucci. She performed a double-blind, placebo-controlled study of overweight or obese children aged 7 to 12 years. They were divided into two groups, one who received oligofructose, 8 g/day, and another group who received placebo for 16 weeks. At the end of 16 weeks, children who consumed prebiotics showed significant weight loss as well as a significant reduction in interleukin 6. Regarding the composition of the intestinal microbiota, it was observed that in the group of those who consumed prebiotics there was a significant increase in *Bifidobacterium* spp. and decreases of *Bacteroides vulgatus* [99].

Administration of 100 g/day of choline and fructooligosaccharide (FOS) to mice resulted in a reduction in liver fat, being effective in reducing hepatic steatosis [100]. Takai and co-workers also found that supplementation with FOS in monosodium glutamate-injected mice reduced hepatic steatosis, inflammatory cell infiltration and hepatocyte ballooning [51].

## 5. The Biotics Family–Future Perspectives in Metabolic Diseases

### 5.1. Next Generation of Probiotics and Prebiotics

In recent years, thanks to the continuous development of high-performance sequencing technology, researchers have gained a clearer understanding of gut microorganisms and the accuracy of species resolution is also increasing, and a mysterious “dark matter” harbored in the gut is being revealed step by step. More research shows a positive association between *Oscillospira* and low fats, leanness and human health, as well as its ability to produce short-chain fatty acids, especially butyrate. If *Oscillospira* shows potential for development as a probiotic, it can be considered a favorable candidate for the next generation. In the future, more studies are needed to confirm the efficacy of *Oscillospira* in different diseases, and if the pure culture technology of this organism can be overcome, it will greatly improve the process of its development and application [101].

Another candidate is represented by *Dysosmobacter welbionis J115T*, which is a new bacterium isolated from the human intestine and which produces butyrate. Tiphaine Le Roy and his team conducted a study that highlighted the beneficial effects of *Dysosmobacter welbionis J115T* in the case of obesity and diabetes. They identified approximately 65% (62.7–69.8%) of the new bacteria in healthy people. The genus *Dysosmobacter* correlated negatively with body mass index, fasting glucose and glycated hemoglobin. Additionally, administration of *D. welbionis J115T* to mice protected against brown adipose tissue inflammation. These observations suggest that *Dysosmobacter welbionis J115T* is also a strong candidate for the development of the next-generation beneficial bacteria that target obesity and associated metabolic diseases [102].

*Clostridium tyrobutyricum L319* is a bacterium producing short-chain fatty acids that was isolated from Grana Padano cheese. Zhihan Yang et al. highlighted the qualities of this bacterium to be considered a probiotic. Thus, this bacterium shows significant hydrophobicity in acidic conditions and resistance to gastric juice. In addition, it also withstands harsh environmental conditions such as low or high temperatures. Another quality of this bacterium is represented by its ability to ferment prebiotics to produce SCFA. All these properties make it a promising candidate for future use as a probiotic [103].

As far as prebiotics are concerned, a future candidate is represented by Galactosyl-β1,4-L-rhamnose (Gal-β1,4-Rha). This selectively promoted the growth of *Bifidobacterium*. In vivo, administration of B. infantis and Gal-β1,4-Rha attenuated weight loss associated with *Clostridium difficile* infection in mice. Additional studies are still needed, but current data are promising regarding the use of this prebiotic in various pathologies [104].

### 5.2. Postbiotics

Although probiotics have many benefits for the health of the human body, there are doubts about their functionality and practical use, due to the side effects they can cause, as well as the possibility of a horizontal gene transfer of a virulence gene from a pathogenic bacterium in the gastrointestinal tract. Due to these doubts, recent research has shown that the benefit of using non-viable microorganisms is similar to that of using viable microorganisms [105,106]. The term *postbiotic* is based on the fact that beneficial effects of the gut microbiota are mediated by the secretion of various metabolites. This term was also limited to metabolites or cell-free supernatants and soluble factors secreted by living bacteria. The postbiotic is also called metabiotic and represents structural components of microorganisms or their metabolites/signaling molecules that can optimize the physiological functions of the host. The main postbiotics are organic acids, short chain fatty acids (SCFA), tryptophan, cell-free supernatants, exopolysaccharides, enzymes, cell wall fragments and bacteriocins [105,107]. Due to the high diversity of substances classified as postbiotics, the mechanisms of action are complex and not fully understood. Postbiotics shows (1) immunomodulatory effects, (2) antitumor effects, (3) antimicrobial effects and (4) antiatherosclerotic effects [107].

#### Postbiotics and Metabolic Diseases

The concept of postbiotics is a new one, so studies on their clinical applications are still in their infancy. Kun-Ho Seo and colleagues highlighted the synergistic anti-obesity effect of heat-killed lactic acid bacteria (HLAB) and prebiotic polyphenol-rich wine grape seed flour (GSF). Thus, the researchers administered to the mice a diet high in fat and fructose (HFFrD) with 5% microcrystalline cellulose (CON), HGGrD with 2.5% GSF, HFFrD with HLAB and HFFrD combined with GSF and HLAB for 8 weeks. At the end of the study, it was observed that in mice receiving GSF and HLAB, there were significant reductions in weight gain induced by a diet high in fat and fructose [108].

Due to the fact that postbiotics are still new preparations, studies and research on their effects on metabolic diseases are still in their infancy. An example of a study is the one conducted by Nakamura and his collaborators on a group of 200 overweight participants with a body mass index between 25–30 kg/m^2^. Overweight participants were divided into two groups: one received 200 mg of *Lactobacillus amylovorus CP1563* and the other group received a placebo for 12 weeks. At the end of these periods, it was observed in the test group a reduction of body fat and lipid profile with decreased triglycerides and total cholesterol [109]. Another study highlighting the link between postbiotics and obesity is conducted by Tomonori Sugawara and colleagues. This study included 169 individuals with a body mass index between 25 and 29.9 kg/m^2^ who either ingested for 12 weeks drinks with fragmented *Lactobacillus amylovorus CP1563 (CP1563)* containing 10-hydroxyoctadecanoic acid (10-HOA) or placebo drinks. At the end of the period, a reduction in abdominal body fat was observed in the group of those who consumed drinks with paraprobiotics [110].

The effect of drinking the *Lactobacillus casei 01* inactivated by ohmic heating was demonstrated both in vitro where it had a hypoglycemic action by inhibiting alpha glucosidase and alpha amylase, and in vivo, in 15 healthy subjects who consumed either bread and probiotics drinks, bread and postbiotics whey drink, or just bread. Those who consumed bread with whey paraprobiotic drink had lower postprandial glycemic value compared to the other two groups. Therefore, postbiotics whey drink may be considered a therapeutic alternative for those with type 2 diabetes mellitus, but to be sure, additional studies are needed [111].

A study in mice highlights the possible beneficial effects of postbiotics on the development of type 2 diabetes mellitus, namely the long-term additional effect of a fermented milk-free food (FFP) in the Zuker Diabetic and Fatty (ZDF) rats’ type 2 diabetes model. At the end of the study, it was observed that supplementation with FFP produced an improvement in glucose homeostasis. Also, in mice with FFP, a reduction in intestinal glucose absorption was observed [112].

The most common postbiotics come from *Lactobacillus* and *Bifidobacterium* species. The strains of these species have beneficial effects in their inactivated form. An example is the case of *Bifidobacterium breve M-16V*. Sugahara and colleagues have shown that the use of both live and inactivated *B. breve M-16* has immune-modulating effects that inhibited the production of proinflammatory cytokines in spleen cells [113].

Being still in its infancy, studies on the beneficial effects of postbiotics on metabolic diseases are largely performed in animals. Ali Osman and colleagues emphasized the role of the lipolytic postbiotic *Lactobacillus paracasei* on the metabolism of this lipid, as well as the possibility of being a substitute for atorvastatin. This postbiotic was administered to rats on a high-fat diet (HFD) and was compared to rats receiving atorvastatin for 9 weeks. The postbiotic decreased serum lipid and triglyceride levels. The high uptake activity of DPPH (1,1-diphenyl-2-picrilhydrazyl) and ABTS [2,2′-azino-bis (3-ethyl benzothiazolin-6-sulfonic acid)] as well as the high activities of antioxidant enzymes, such as be superoxide dismutase (SOD), catalase (CAT) and glutathione peroxidase (GSH-px) of postbiotic *Lactobacillus paracasei* (cell-free extract), may explain the effectiveness of this postbiotic in its fight against metabolic syndrome [114].

Another study where the beneficial effect of postbiotics is highlighted is the one conducted by Jaehoon Lee and his collaborators, where he studied the mechanism of the action of *Lactobacillus plantarum (L-14)* and the results of regular administration on a group of mice that had a high-fat diet. Thus, in his study, 3T3-L1 mouse pre-adipocyte cells and human bone marrow mesenchymal stem cells (hBM-MSC) were treated with *Lactobacillus plantarum L-14* extracts every 2 days and analyzed both at the cellular and molecular level. The L-14 extract was given to mice that had a high-fat diet and compared to a group of mice that did not receive L-14 for a period of 7 weeks. At the end of the study, it was observed that L-14 inhibited 3T3-L1 and hBM-MSC differentiation in mature adipocytes by regulating the AMPK signaling pathway in the early stages of adipogenic differentiation. Weight loss in the group of mice receiving L-14 was also observed [115].

Another postbiotic that has beneficial effects on obesity and insulin resistance is the bacterial cell wall-derived muramil dipeptide (MDP). This is an insulin sensitizing postbiotic. Its administration reduced fat inflammation and glucose intolerance in obese mice. MDP has also been shown to reduce hepatic insulin resistance [116].

## 6. Conclusions

There is a close link between metabolic diseases and the intestinal microbiota, and it has been shown that the latter plays an important role in the development of obesity, type 2 diabetes mellitus and non-alcoholic fatty liver disease. Therefore, it is feasible to say that modeling it through external approaches can bring beneficial effects on the host organism. These approaches are represented by the external administration of biotics family members. The findings and scientific research conducted so far strengthen the idea of the potential impact of family members of biotics, a perspective that could be particularly important in the era of personalized medicine. Knowledge about the biotics family remains limited and further studies are needed to establish the beneficial effects on metabolic diseases. Despite several studies addressing each class of the biotics family currently available, there are still limitations that are related to the number of participants, the duration of treatment or the geographical expansion. Therefore, future studies in this field should seek to correct these disadvantages in order to have the most diverse results possible.

## Figures and Tables

**Table 1:** Examples of microorganisms used as probiotics (adapted to Gupta et al. [63]).

*Lactobacillus sps.*	*Streptococcus sps.*	*Saccharomyces sps.*	*Bifidobacterium sps.*	Others
*L. acidophilus* *L. reuteri* *L. gasseri* *L. plantarum* *L. casei (rhamnosus)* *L. fermentum* *L. lactis* *L. paracasei*	*S. salivarius subsp. thermophilus* *S. thermophilus*	*S. boulardii*	*B. bifidum* *B. lactis* *B. adolescentis* *B. longum* *B. infantis* *B. breve*	*Propionibacterium freudenreichii* *Bacillus cereus* *Escherichia coli* *Enterococcus*

**Table 2:** Prebiotic sources (adapted to Chudzik et al. [94]).

Source	Prebiotics
Asparagus, chicory, the blue agave plant,	Fructooligosaccharides (FOS)
Soybean	Soybean oligosaccharide (SOS)
Chicory, garlic, asparagus, onion, yacon	Inulin
Milk	Lactulose
Lycopus lucidus herb	Galactooligosaccharides (GOS)
Honey, rice, corn cob	Xylooligosaccharide (XOS)
Palm kernel products	Mannooligosaccharides (MOS)
Cereal grains, seeds, starchy fruits and vegetables	Resistant starch

## Data Availability

This research did not report any data.
